# Evaluation of antimicrobial and antibiofilm properties of proanthocyanidins from Chinese bayberry (*Myrica rubra* Sieb. et Zucc.) leaves against *Staphylococcus epidermidis*

**DOI:** 10.1002/fsn3.1283

**Published:** 2019-11-27

**Authors:** Mingming Zou, Wenyang Tao, Xingqian Ye, Donghong Liu

**Affiliations:** ^1^ National Engineering Laboratory of Intelligent Food Technology and Equipment Zhejiang Key Laboratory for Agro‐Food Processing College of Biosystems Engineering and Food Science Fuli Institute of Food Science Zhejiang University Hangzhou China

**Keywords:** antibacterial, antibiofilm, Chinese bayberry leaves, proanthocyanidins, *Staphylococcus epidermidis*

## Abstract

*Staphylococcus epidermidis* has emerged in recent years as one of the most important opportunistic pathogens owing to its ability to attach to processing surfaces in the food industry. Demands of foodstuffs maintaining microbiological safety and stability enhance the need to develop natural antimicrobial agents as food preservatives. Proanthocyanidins from Chinese bayberry leaves (BLPs) belonging to the class of polyphenols promise to be a potential antibacterial material against bacterial adhesion and biofilm formation. The aim of the present study was to investigate the effects of BLPs on *S. epidermidis* growth and biofilm formation. BLPs possessed antimicrobial activity with MIC and MBC of 320 and 640 μg/ml, respectively. Scanning electron microscopy, transmission electron microscopy, and flow cytometry analysis revealed a loss of the cell structure and function after treatment of BLPs, evidenced by cell membrane hyperpolarization and changes in cellular morphology. BLPs inhibited the biofilm formation by *S. epidermidis* on polystyrene microplates. Atomic force microscopy analysis showed that BLPs could decrease the stiffness and adhesion force of the cell envelope, which might account for the inhibition of biofilm formation. In summary, this study indicated that BLPs have potential to be developed as natural preservatives to control *S. epidermidis* in foods.

## Graphical abstract

A loss of the cell structure and function appeared after treatment of BLPs, evidenced by cell membrane hyperpolarization and changes in cellular morphology. Meanwhile, BLPs could decrease the stiffness and adhesion force of the cell envelope, which might account for the inhibition of biofilm formation.

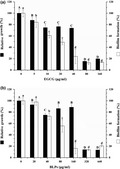

## 1 INTRODUCTION

There has been a growing public concern on food safety due to an increasing occurrence of foodborne illness outbreaks caused by pathogenic and spoilage microorganisms (Tajkarimi, Ibrahim, & Cliver, 2010). *Staphylococcus epidermidis*, a coagulase‐negative staphylococcus, has emerged as one of the most important opportunistic pathogens owing to its ability to attach to processing surfaces in the food industry (Gomes, Teixeira, Cerca, Azeredo, & Oliveira, 2011; Zou & Liu, 2018). Since enterotoxigenic *S. epidermidis* strains were isolated from foodstuffs and ready‐to‐eat meat products, it highlighted the need for studies on the involvement of the biofilm formation by *S. epidermidis* under food‐related conditions (Rall et al., 2010).

Nowadays, chemical additives are commonly used in food products to inhibit microbial growth. However, the drumbeat of concern about the uncontrolled use of chemical preservatives has grown louder in recent years (Witkowska, Hickey, Alonso‐Gomez, & Wilkinson, 2013). Consequently, natural antimicrobial compounds are receiving a good deal of attention for the safety of food products, including extracts of some spices, herbs, and other plants (Tajkarimi et al., 2010).

Among phytochemicals, phenolic compounds have been extensively studied due to their diverse health benefits, mainly as antioxidants, anti‐inflammatory, and antimicrobial agents (Daglia, 2012). Proanthocyanidins (PAs), also known as condensed tannins, were extracted in fruits, bark, leaves, and seeds of many plants (Daglia, 2012). They are oligomeric flavonoids of catechin, epicatechin, and their gallic acid esters (Jagannathan & Viswanathan, 2018). Chinese bayberry (*Myrica rubra* Sieb. et Zucc.) has been cultivated in Southern China for more than 2000 years, and its leaves are luxuriant but always discarded (Zhang, Chen, Wei, Chen, & Ye, 2017). Studies showed that phenolic extracts from bayberry leaves exhibit antimicrobial properties (Li, Han, Chen, & Ye, 2012). However, the effects of phenolic extracts from bayberry leaves on biofilm formation are poorly recognized.

The structure of PAs depends on the nature of flavan‐3‐ol linkages substituted with hydroxyl groups along with aromatic and fused oxetane rings (Jagannathan & Viswanathan, 2018). A special type of PAs from Chinese bayberry leaves was previously identified by our group (Fu et al., 2014; Yang et al., 2011). In comparison with other types of PAs from plants, such as apple, cranberry, or grape seeds, proanthocyanidins from Chinese bayberry leaves (BLPs) contain a simple but potent bioactive unit, that is, epigallocatechin gallate (EGCG); thus, the structural‐activity relationship for BLPs might be clearer than PAs from other plants (Zhang et al., 2017).

The current study investigated the ability of BLPs on inhibiting bacterial growth and biofilm formation by *S. epidermidis*, which was isolated from a milk powder processing factory. The mechanisms of antimicrobial and antibiofilm actions of BLPs were also investigated by a series of techniques, including scanning electron microscopy (*SEM*), transmission electron microscopy (TEM), flow cytometry analysis (FCM), and atomic force microscopy (AFM) nanoindentation.

## 2 MATERIALS AND METHODS

### 2.1 Polyphenols

The BLPs used in this study were obtained according to our previous studies (Fu et al., 2014; Yang et al., 2011; Zhang et al., 2016). A stock solution was prepared by dissolving 25 mg BLPs powder in 1 ml sterile distilled water, and then, the solution was filtered through a 0.22 μm‐pore membrane filter. EGCG was used as a positive control to evaluate the antibacterial and antibiofilm capabilities of BLPs. EGCG (Shanghai Yuanye Biotechnologies Co.; purity of approximately 90%) was also dissolved in sterile distilled water at a concentration of 5 mg/ml and was filter sterilized as above. The final concentrations of BLPs ranged from 20 to 2,560 μg/ml, and the final concentrations of EGCG ranged from 5 to 640 μg/ml.

### 2.2 Bacteria and growth conditions

*Staphylococcus epidermidis* isolated from a milk powder processing factory located in northeastern China was cultured in tryptic soy broth (TSB, Hopebio) at 37°C for 18 hr to achieve the stationary phase (Zou & Liu, 2018). Cells were harvested by centrifugation (2,350 × *g*, 4°C for 10 min） and then washed twice by sterile phosphate‐buffered saline (PBS, pH 7.4) solution and resuspended in fresh sterile TSB (a final concentration about 10^9^ CFU/ml).

### 2.3 Determination of MICs and MBCs

Minimal inhibitory concentrations (MICs) and minimal bactericidal concentrations MBCs of BLPs against *S. epidermidis* were determined using the twofold microplate dilution assay as described by Grenier, Chen, Ben Lagha, Fournier‐Larente, and Morin (2015) with minor modifications. A suspension of stationary phase bacteria in TSB (approximately 10^6^ CFU/ml) was added to each well of the 96‐well polystyrene microplates (Costar^®^ 3599, Corning Life Science). MICs of BLPs were determined as the lowest concentration at which no turbidity can be observed after incubation at 37°C for 24 hr. To determine MBCs, 100 μl of each well showing no visible growth of *S. epidermidis* was spread on tryptone soya agar (TSA, Hopebio) plates. MBCs of compounds were determined as the lowest concentration at which no colony formation occurred after incubation at 37°C for 24 hr. All tests were performed in triplicate.

### 2.4 Detection of viable and sublethally injured cells

To estimate the amounts of sublethally injured cells, *S. epidermidis* cells treated with BLPs at the MIC at 37°C for 24 hr were spread on nonselective and selective plates. Then, these plates were incubated at 37°C for 24 hr. In this study, the nonselective medium was TSA without additional NaCl, while the selective medium was TSA containing 5% NaCl. Stress‐induced sublethal *S. epidermidis* were counted by obtaining the differences in colony counts between the nonselective and selective plates.

### 2.5 Determination of MBICs

Minimal biofilm formation inhibitory concentrations (MBICs) were determined. *S. epidermidis* was subjected to a biofilm assay using 96‐well polystyrene microplates for the examination of biofilm prevention, as described previously (LaPlante, Sarkisian, Woodmansee, Rowley, & Seeram, 2012). The plates were incubated statically for 24 hr at 37°C to allow biofilm formation, followed by the crystal violet staining method (Zou & Liu, 2018). MBICs of compounds were determined as the lowest concentration at which no detectable biofilm formation occurred.

### 2.6 *SEM* assay

*SEM* was employed to confirm the changes in surface morphology according to Matijasevic et al. (2016). Briefly, the cell resuspensions were treated with polyphenols at the MIC level. Control (untreated bacteria) and treated samples were incubated for 2 hr at 37°C and then harvested by centrifugation at 2,350 × *g*, 4°C for 10 min, followed by immobilization with 2.5% glutaraldehyde overnight at 4°C. These samples were dehydrated by a graded series of ethanol (30%, 50%, 70%, 80%, 90%, 95%, and 100%), coated with gold–palladium, and observed in a Hitachi Model SU‐8010 *SEM* (Hitachi, Ltd.).

### 2.7 TEM assay

Ultrastructural damage of the bacterial cells was evaluated using TEM. Bacterial samples were prepared and dehydrated as mentioned in the *SEM* assay. Then, these samples were placed in a 1:1 acetone–Spurr resin mixture for 1 hr; a 1:3 acetone–Spurr resin mixture for 3 hr; and the absolute Spurr resin overnight at room temperature. Next, samples were sectioned in a LEICA EM UC7 ultratome. Finally, these sections were stained with uranyl acetate and alkaline lead citrate prior to observation in a Hitachi Model H‐7650 TEM (Hitachi, Ltd.).

### 2.8 Flow cytometric analysis for cell membrane permeability

Bacterial samples were prepared as mentioned in the *SEM* assay, followed by measurement using the LIVE/DEAD *Bac*Light™ Bacterial Viability Kit (L‐7012, Invitrogen). Briefly, one milliliter of *S. epidermidis* resuspensions was incubated with BLPs or EGCG at the MIC level, respectively. Control (untreated bacteria) and treated samples were incubated for 2 hr at 37°C. After incubation, cells were harvested by centrifugation at 2,350 × *g*, 4°C for 10 min and washed thrice with PBS. Then, these samples were diluted 1:100 in filter‐sterilized ddH_2_O to reach a final density of 1 × 10^6^ CFU/ml. Last, one milliliter of each sample was mixed with 3 μl PI and 3 μl SYTO 9 and incubated at room temperature in the dark for 15 min. Stained samples were assayed in a Gallios flow cytometer equipped with a fully functional double laser and eight detectors (Beckman Coulter Inc.), and a total of 20,000 events were recorded. Data were analyzed using the Kaluza software package (Beckman Coulter Inc.). A heat‐treated bacterial suspension (80°C/20 min) was analyzed as a positive control to check the applicability of the staining protocol for the sample analysis.

### 2.9 Flow cytometric analysis for cell membrane potential

The effects of BLPs on the cell membrane potential of *S. epidermidis* were measured using the *Bac*Light™ Bacterial Membrane Potential Kit (B34950, Invitrogen) as mentioned by Mora‐Pale et al. (2015). Briefly, bacterial samples were prepared as mentioned in the *SEM* assay. Meanwhile, an additional sample (untreated bacteria) was prepared as a depolarized control by mixing with 10 µl of 500 µM carbonyl cyanide 3‐chlorophenylhydrazone (CCCP). One milliliter of each treated sample was mixed with 10 μl of 3 mM DiOC_2_(3) and incubated at room temperature for 15 min. Last, samples were assayed in the Gallios flow cytometer and a total of 20,000 events were recorded. Detection mode and data analysis were performed as described above.

### 2.10 AFM analysis and force measurements

The effects of BLPs on the structural, adhesive, and mechanical properties of the cell envelope were measured by AFM based on Mularski, Wilksch, Hanssen, Strugnell, and Separovic (2016). Briefly, bacterial samples were prepared in the same manner as described in the *SEM* assay at a concentration of MBIC. A cell suspension (10 μl) was deposited onto a piece of mica plate (10 × 10 mm, Beijing Zhongjingkeyi Technology Co., Ltd) and dried naturally. The surface micrographs were imaged at room temperature in the air with a tapping mode by AFM (Cypher S, Oxford Instruments). A nominal resonance frequency of 300 kHz and a 50 N/m spring constant were applied. NanoScope analysis software (version 1.5, Bruker) was used for image manipulation. In addition, an XE‐70 AFM (XE‐70, Park Scientific Instruments) was used for adhesion force measurements using a cantilever (Si_3_N_4_) with a scan rate of 1 Hz and a nominal spring constant of 0.08 N/m. XEI data processing software (version 1.8.0, Park Systems Corporation) was used for image manipulation.

### 2.11 Statistical analysis

Unless indicated otherwise, all data were presented as means ± standard deviations (*SD*) based on three independent experiments. The data from all assays were compared using one‐way analysis of variance (ANOVA) by applying Tukey's test with all calculations carried out using IBM SPSS Statistics 20.0 software (IBM Inc.), and the statistical significances were achieved when *p* < .05.

## 3 RESULTS AND DISCUSSION

*Staphylococcus epidermidis* plays an influential role in creating the pathogenic biofilm. The present study describes antimicrobial and antibiofilm activities and mechanisms of BLPs against *S. epidermidis*. Two polyphenols with a different mean degree of polymerization (mDP) were compared: BLPs (mDP at about 7.3 ± 0.1) and EGCG (positive control, monomer).

### 3.1 BLPs antibacterial activity against planktonic *S. epidermidis* cells

As reported in Table 1, the MIC and MBC values of BLPs were 320 μg/ml and 640 μg/ml, respectively. The viable and sublethal cells of *S. epidermidis* incubated with BLPs at the MIC are shown in Table S1 (Appendix S1). The survivors on the selective medium supplemented with 5% NaCl could be assigned as undamaged cells, while those on the nonselective medium included both undamaged and sublethally damaged cells. As shown in Table S1, cells incubated with EGCG at the MIC cannot be cultured on the selective media. However, the number of sublethal cells after BLPs treatment was negligible; therefore, there was no sublethal damage caused by BLPs treatment.

**Table 1:** Biological activity of EGCG and BLPs (μg/ml) against *Staphylococcus epidermidis*

Compounds	MIC	MBC	MBIC
EGCG	80	160	40
BLPs	320	640	160

Bacterial cells treated with EGCG at the MIC were unable to recover viability on the selective media, whereas those treated with BLPs at the MIC were able to recover viability overall on the same media. Adaptation of bacteria by exposure to sublethal levels of some stresses was well documented (Fernández et al., 2018). Exposure of bacteria to sublethal concentrations of some inhibitory compounds may induce the expression of stress shock proteins and lead to cross‐protection against a range of apparently unrelated challenges including antibiotics (McMahon, Xu, Moore, Blair, & McDowell, 2007). In the present study, BLPs‐adapted *S. epidermidis* exhibited enhanced survival rate when exposed to the selective medium with a high concentration of NaCl. Additionally, cells were induced to a multicellular phenotype (Figure 2C‐c), which might be a survival strategy for staphylococci grown under the influence of different treatments as described earlier (Bikels‐Goshen, Landau, Saguy, & Shapira, 2010). Meanwhile, a significant increase in the cell wall thickness of *S. epidermidis* exposed to BLPs was found, which agreed with the phenomenon of staphylococci in the previous study (Bikels‐Goshen et al., 2010). These results showed that exposure to BLPs presumably induced cell clustering and an increase in cell wall thickness, which in turn might contribute to the higher osmotic tolerance.

Although the inhibitory effect of BLPs against *S. epidermidis* was less benign than that of cranberry extracts with a MIC of 160 μg/ml as reported by LaPlante et.al (2012), BLPs showed more favorable bactericidal activity with a MBC of 640 μg/ml (Table 1) than that of cranberry extracts with a MBC range from 1,250 to 5,000 μg/ml. As put forward by Mostafa et al. (2018), the difference in the MIC of plant extracts can be due to extensive variation in their method of extraction, constituents, and structural nature of their constituents. These results suggested that BLPs were promising and effective compounds for antibacterial applications.

### 3.2 Morphological and ultrastructural changes of *S. epidermidis*

The antibacterial mode of action of BLPs against *S. epidermidis* in the present was evidenced in two ways. Firstly, microscopies were applied to identify the morphological appearance, ultrastructure, and topography of *S. epidermidis*. Secondly, FCM was used to show that polyphenols appear to change the membrane potential, damage the integrity, and cause functional disorder of the bacterial cell membrane.

**Figure 1:**
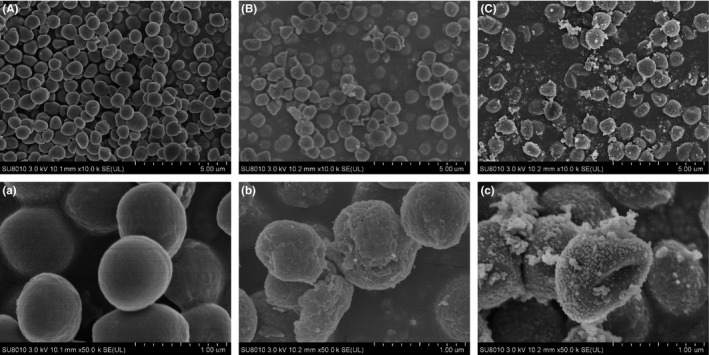
Scanning electron microscopy analysis of *Staphylococcus epidermidis*. Panel A‐a: Untreated bacteria. Panel B‐b: Bacteria treated (2 hr) with epigallocatechin gallate (80 μg/ml). Panels C‐c: Bacteria treated (2 hr) with BLPs (320 μg/ml)

**Figure 2:**
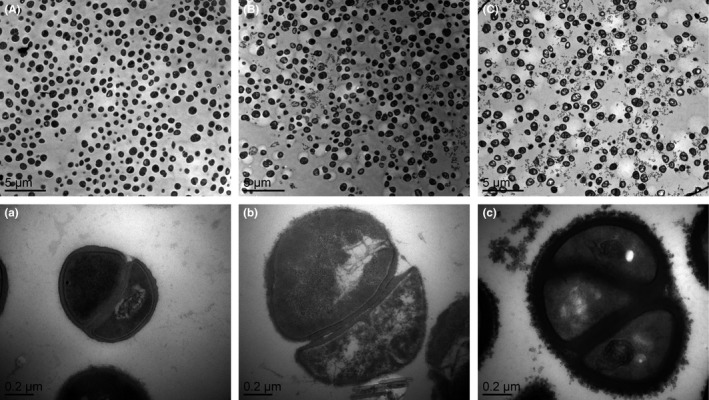
Transmission electron microscopy analysis of *Staphylococcus epidermidis*. Panel A: Untreated bacteria. Panel B: Bacteria treated (2 hr) with epigallocatechin gallate (80 μg/ml). Panels C: Bacteria treated (2 hr) with BLPs (320 μg/ml)

Morphological and ultrastructural changes of *S. epidermidis* incubated with BLPs at the MIC were analyzed via *SEM* (Figure 1) and TEM (Figure 2). Figure 1A‐a and B‐b clearly showed cell damage when *S. epidermidis* was treated with EGCG compared to the control cells. The *S. epidermidis* cells grown in the presence of BLPs at the MIC were abnormally shaped (Figure 1C‐c), and particles of white precipitates can be identified on the surfaces of bacterial cells. Moreover, compared with control cells (Figure 2A‐a), the ultrastructure of *S. epidermidis* cells was obviously affected by BLPs treatment at the MIC (Figure 2C‐c). A significant increase in the cell wall thickness of *S. epidermidis* exposed to BLPs was found.

As is well known, the cell membrane is an active structure that acts as a barrier between the inner and outer portions of the cell and plays a key role in maintaining optimal internal conditions for metabolism and energy transduction. Many studies have suggested that phenolic compounds, such as syringic acid, primarily target the cytoplasmic membrane and retarded bacterial growth (Cui et al., 2012). In the present study, BLPs contain unique EGCG as its terminal unit and most of its extension units, with an mDP of 7.3 ± 0.1 (Zhang et al., 2017). The previous studies revealed that PAs with lower mDP demonstrated better bioactivity properties (Zhang et al., 2016). Thus, it is reasonable that BLPs showed less efficient antibacterial properties with a comparison to EGCG.

Changes in the morphology of *S. epidermidis* treated with BLPs at the MIC for 2 hr were slightly different from those caused by EGCG, see Figure 1B‐b and C‐c. The presence particles of white precipitates in association with the bacterial cells in Figure 1C‐c suggested that BLPs targeted to alter the cell wall and/or the cell membrane, while the EGCG not. TEM analysis confirmed this hypothesis, showing that the ultrastructure of bacteria treated with BLPs had visible changes (Figure 2C‐c) compared with the control cells (Figure 2A‐a), as there was a significant increase in the cell wall thickness. This could be associated with an accumulation of cross‐linking between BLPs and the cell envelope. Hence, BLPs probably bound to the peptidoglycan layer and/or the cytoplasmic membrane of *S. epidermidis*, causing damage to the cell membrane and loss of the membrane permeability control, resulting in the cell membrane dysfunction and bacterial growth inhibition.

### 3.3 Membrane integrity and potential changes of *S. epidermidis* by FCM

**Figure 3:**
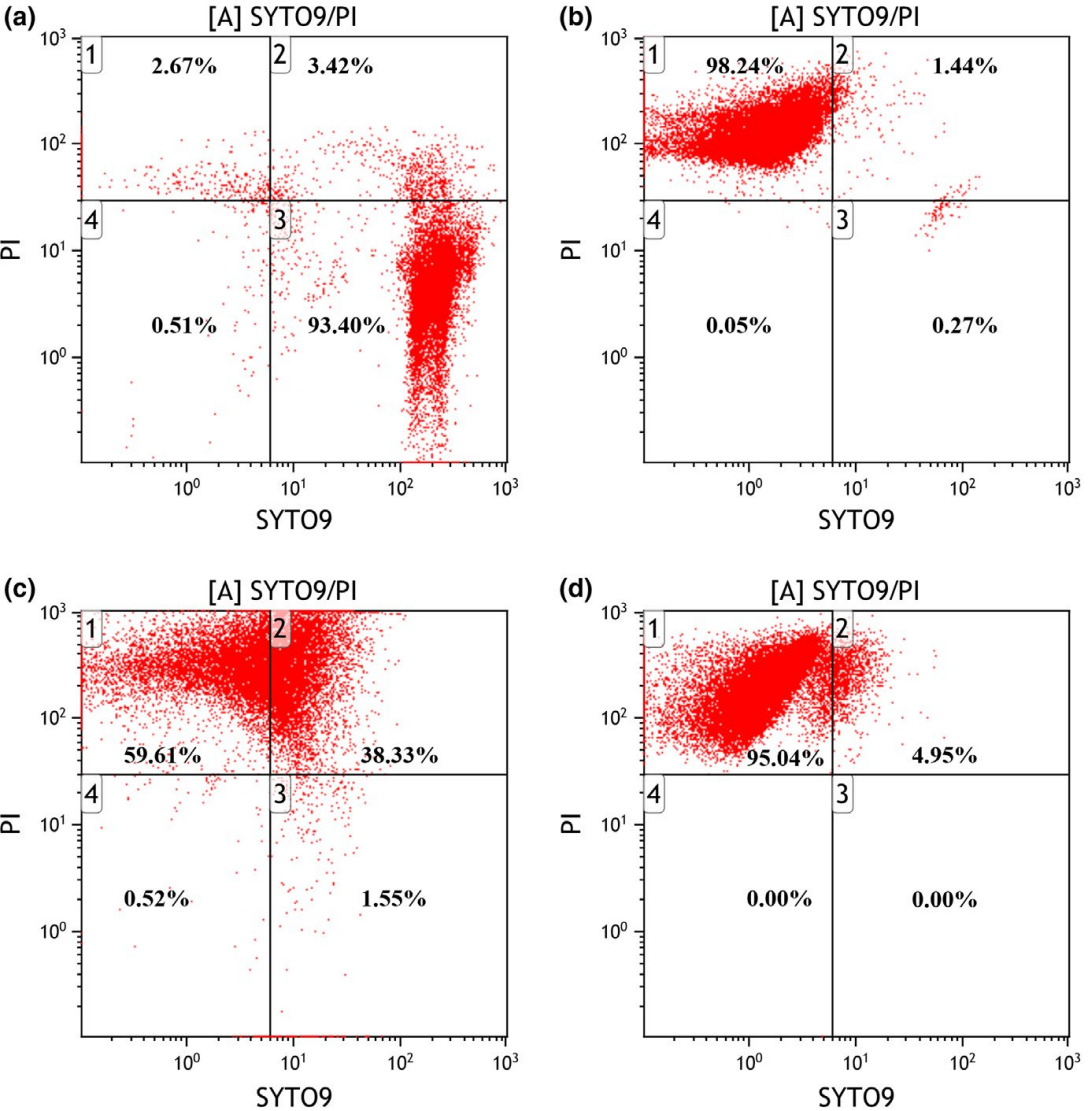
Fluorescence dot plots of *Staphylococcus epidermidis* in response to double staining with SYTO9 and PI. Panel A: Untreated bacteria. Panel B: Heat‐treated cells (20 min at 80°C). Panel C: Bacteria treated (2 hr) with epigallocatechin gallate (80 μg/ml). Panels D: Bacteria treated (2 hr) with BLPs (320 μg/ml)

**Figure 4:**
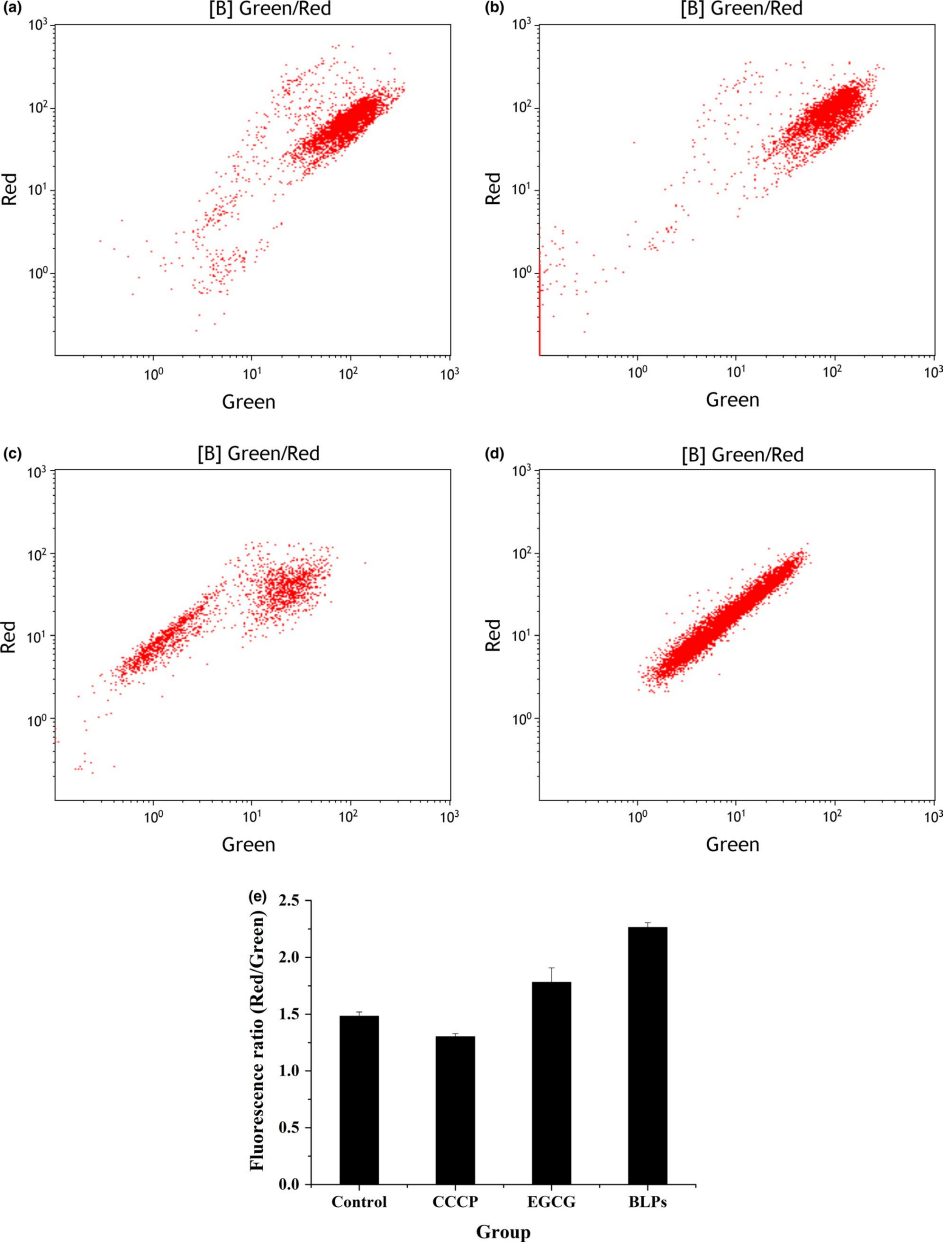
Effects of epigallocatechin gallate (EGCG) and BLPs on the membrane potential of *Staphylococcus epidermidis*. Panel A: Unstained bacteria. Panel B: Control bacteria stained with carbonyl cyanide 3‐chlorophenylhydrazone (5 μg/ml). Panel C: Bacteria treated (2 hr) with EGCG (80 μg/ml). Panels D: Bacteria treated (2 hr) with BLPs (320 μg/ml). Panel E: Red/green ratios were calculated using population mean fluorescence intensities

Figure 3 and Figure 4 showed the effects of BLPs on the cytoplasmic membrane of *S. epidermidis*. The fluorescence dot plots were based on two control populations, one of the untreated cells emblematic of intact/live cells (Figure 3A) and the other of heat‐treated cells emblematic of permeabilized/damaged cells (Figure 3B) (Witkowska et al., 2013). As shown in Figure 3A, untreated cells were stained as SYTO 9 positive and mainly located in lower and upper right quadrants, and their viability was confirmed by plate count on TSA plates. For control cells inactivated by heating (Figure 3B), populations with permeabilized/damaged membranes were stained as PI positive and located in the upper left quadrant. Cells in the upper right quadrant stained as both SYTO 9 and PI positive, which might indicate cells with damaged membranes allowing penetration of PI into the cell interior. When treated with EGCG at the MIC, a clear shift from green fluorescence toward red fluorescence was observed, with 59.61% of permeabilized/damaged cells located in the upper left quadrant and 38.33% of cells located in the upper right quadrant in Figure 3C. Cells treated with BLPs at the MIC differed significantly from that of untreated cells with the appearance of 95.04% of permeabilized/damaged cells located in the upper left quadrant (Figure 3D).

Moreover, changes of bacteria cytoplasmic membrane permeability by BLPs at the MIC were also verified by using the membrane potential sensitive dye DiOC_2_(3), which exhibits green fluorescence in all bacterial cells. The red‐versus‐green fluorescence dot plots of *S. epidermidis* cells, including unstained control bacteria, control bacteria stained with CCCP, bacteria treated with EGCG and BLPs at the MIC, were shown in Figure 4A‐D. BLPs caused cell membrane hyperpolarization, as evidenced by the noticeable increase in red‐versus‐green fluorescence (Figure 4E). As well known, the fluorescence shifts toward red emission when cytosolic dye concentrations become higher due to the larger membrane potentials (Yusook, Weeranantanapan, Hua, Kumkrai, & Chudapongse, 2017).

The PI is widely used as a damage marker, which is excluded by cells with intact membranes but can enter cells with compromised membranes (Liu, Xia, Jiang, Yu, & Yue, 2018). In the present study, bacterial cells stained as PI positive had permeabilized/damaged membranes and appeared to retard growth. On the other hand, BLPs at the MIC caused significant hyperpolarization of the cytoplasmic membrane of *S. epidermidis*. Hyperpolarization has been reported as an important type of membrane damage (Liu et al., 2018). Combined with the plate count data, it is implied that BLPs at the MIC permeabilized the bacterial cytoplasmic membrane to retard the growth of bacteria.

### 3.4 Effects of BLPs on biofilm formation, topography, and nanomechanical properties of the cell envelope of *S. epidermidis*

**Figure 5:**
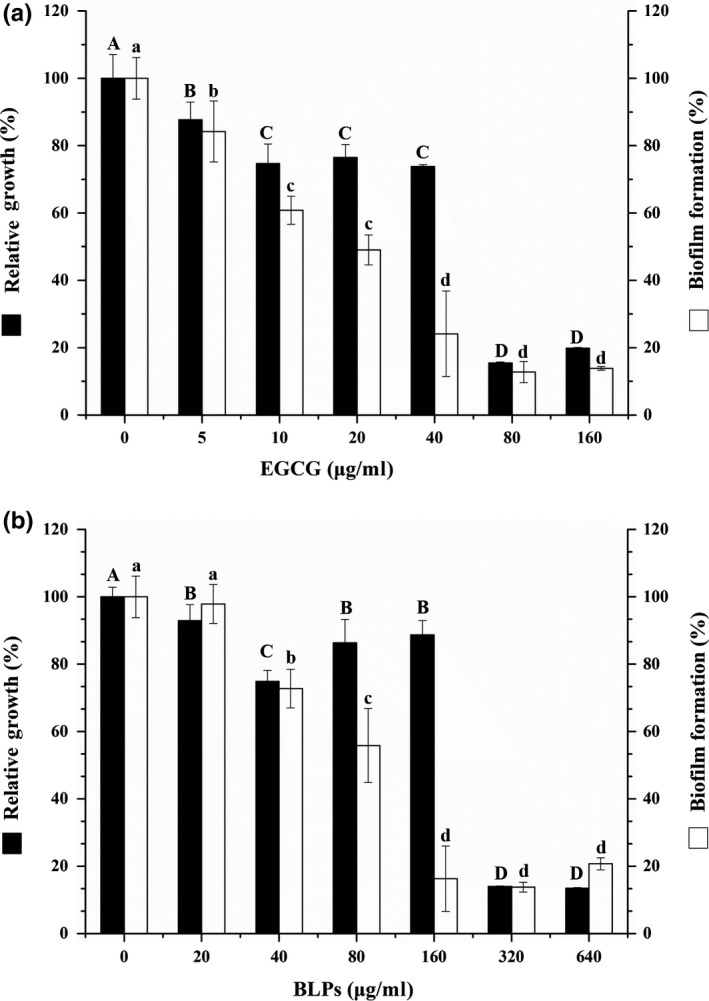
Effects of epigallocatechin gallate and BLPs on the growth of and bacterial biofilm formation by *Staphylococcus epidermidis*. A value of 100% was assigned to growth and bacterial biofilm formation obtained with *S. epidermidis* in the absence of antibacterial material

The inhibition of *S. epidermidis* biofilm formation by BLPs was evaluated by crystal violet staining following growth in a 96‐well microplate. When *S. epidermidis* grew in the presence of BLPs, the specific antibiofilm effect was highly dose‐dependent (Figure 5B). When added at a concentration of 160 μg/ml (MBIC), BLPs reduced biofilm formation by 83.72% without growth inhibition. Biofilm formation decreased with increasing BLPs concentration (320 and 640 μg/ml), due to the growth inhibition effect caused by BLPs.

**Figure 6:**
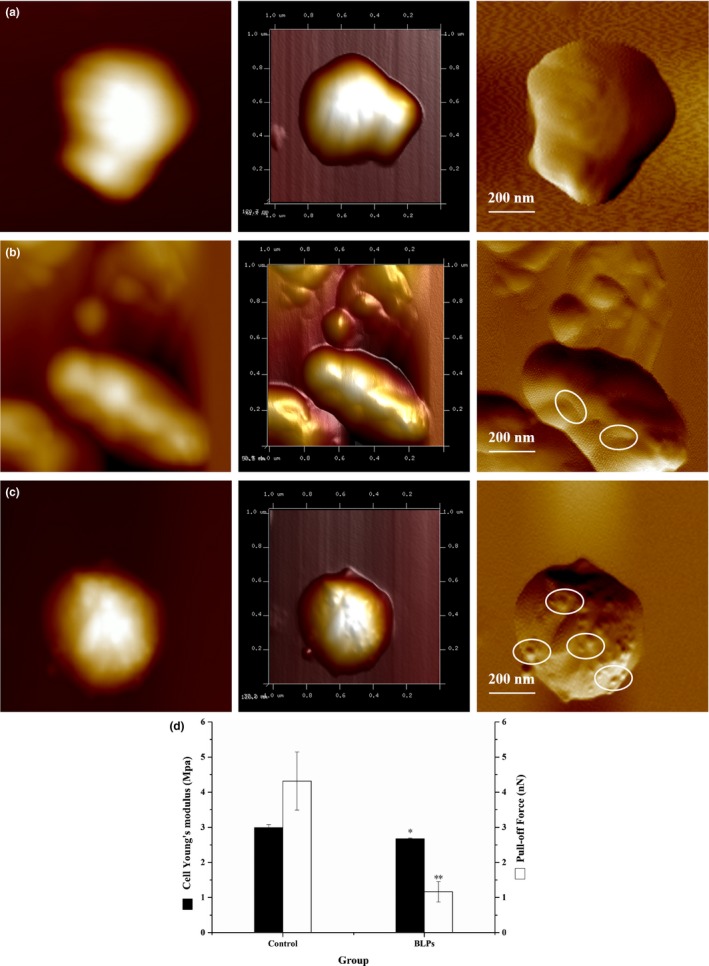
Atomic force microscopy analysis of *Staphylococcus epidermidis* cells. Panel A: Untreated bacteria. Panel B: Bacteria treated (2 hr) with epigallocatechin gallate (40 μg/ml). Panels C: Bacteria treated (2 hr) with BLPs (160 μg/ml). From left to right: two‐dimensional, three‐dimensional, and topological images. Panel D: The changes of stiffness (Young's modulus) and adhesion force of *S. epidermidis* cells treated by BLPs at the Minimal biofilm formation inhibitory concentrations. *Significantly different from the control (*p* < .05). **Significantly different from the control (*p* < .01)

AFM was used to observe topological changes of the cell envelope of *S. epidermidis* when treated with BLPs at the MBIC. As shown in Figure 6A, untreated *S. epidermidis* cells had smooth surfaces with the average surface roughness of 0.83 ± 0.02 (*n* = 5, Table S2), which is in line with Gram‐positive bacteria visualized by this technique (Yang et al., 2010). When treated with EGCG at the MBIC for 2 hr, cells became wrinkled leaving some debris around the cells (Figure 6B), which resulted in a higher surface roughness value of 15.00 ± 7.20 (*n* = 5). Meanwhile, the cells’ average height was decreased to 61.98 ± 16.14 nm (*n* = 5), which was significantly lower than that (146.59 ± 22.26 nm, *n* = 5) of untreated cells (*p* < .05). However, the treatment with BLPs at the MBIC after 2 hr induced the surface to aggregate with the highest surface roughness (45.53 ± 4.15, *n* = 5), while the cells’ average height was increased to 152.39 ± 12.00 nm (*n* = 5) (Figure 6C).

Since it is difficult to clarify the antibiofilm mode of action of BLPs against *S. epidermidis* only based on the morphological data, the approaching part (trace) of the AFM curves was used to calculate the nanomechanical properties of the cell envelope before and after the BLPs treatment. Young's modulus of the point on the cell surface was determined by the Hertz model as previously described (Perni, Preedy, Landini, & Prokopovich, 2016). The changes of stiffness (Young's modulus) and adhesion force of *S. epidermidis* cells treated with BLPs at the MBIC were shown in Figure 6D. Young's modulus of *S. epidermidis* cells treated with BLPs was 2.67 ± 0.02 MPa, which notably reduced compared with that of the native *S. epidermidis* (2.99 ± 0.09 MPa). The tip–cell‐surface adhesion force of *S. epidermidis* cells treated with BLPs was 1.16 ± 0.29 nN, significantly lower than that of the native *S. epidermidis* (4.31 ± 0.83 nN) (*p* < .05).

The ability of *S. epidermidis* to form biofilms plays a key role in food contamination. As is well known, bacteria cells embedded in biofilms are more resistance to both mechanical removal and antimicrobial agents than planktonic cells (Donlan & Costerton, 2002). Thus, compounds that prevent biofilm formation can be used to prevent foodborne illness outbreaks. Young's modulus is one of the mechanical parameters describing the relation between a nondestructive load and resultant deformation of a material. In other words, Young's modulus is sensitive to the internal structural details of heterogeneous materials. Thus, Young's modulus can be used as a probe of hierarchical structure–property relationships (Jin et al., 2010). Results in the present study demonstrated that there is significant variation in bacterial mechanical properties, including Young's modulus and adhesion forces of the bacterial surface before and after the BLPs treatment at the MBIC. The damage to the cell surface was suspected to be the major causes of the decreases in Young's modulus (Jin et al., 2010). As shown in Figure 6C and Table S2, the value of the cell surface roughness increased induced by BLPs suggested accumulation of cross‐linking between BLPs and the cell surface, which may change the permeability of the bacterial cell membrane and lead to the membrane damage. Thus, it was implied that BLPs could damage the structure of the cell wall and/or membrane, resulting in a decrease in Young's modulus. Additionally, there was a noticeable decrease in the adhesion force of the cell surface, which may result in the inhibition of bacterial biofilm formation. In the previous study, Young's modulus has been identified as an important factor in a broad range of biological processes, such as cell differentiation, cell deformability, and cell migration (Guo, Xia, Sandig, & Yang, 2012). Therefore, it was implied that the adhesion force of *S. epidermidis* cells decreased with the reduction of Young's modulus. Taken together, all the AFM morphological and mechanical data indicated that the antibiofilm mode of action of BLPs against *S. epidermidis* at the MBIC was related to the reduction of Young's modulus and cell adhesion force.

## 4 CONCLUSION

For the first time, we reported the antibacterial and anti‐adherence activities of BLPs against *S. epidermidis*. The mode of action of BLPs was attributed to an accumulation of cross‐linking between BLPs and the cell surface. The corresponding membrane damages were observed using *SEM* and TEM and evidenced by cell membrane hyperpolarization and LIVE/DEAD assay. Besides, the elasticity of the cell surface was mapped by AFM to assess the effect of BLPs on the mechanical properties of the *S. epidermidis* cell surface. Young's modulus value and cell adhesion force of the cell envelope both decreased due to BLPs treatment; thus, the biofilm formation was inhibited. The present data suggested that BLPs were a special and valuable resource of bioactive compounds that exert potent antibacterial and antibiofilm effects. Future studies should set up to evaluate the antibacterial and antibiofilm effects of BLPs in food systems and make it a promising novel material in the food industry.

## CONFLICT OF INTEREST

The authors declare that they have no conflict of interest.

## ETHICAL APPROVAL

Human or animal testing is unnecessary in our study.
