# Supplementary material for: Evaluation of antimicrobial and antibiofilm properties of proanthocyanidins from Chinese bayberry (Myrica rubra Sieb. et Zucc.) leaves against Staphylococcus epidermidis
Source: Food Sci Nutr. 2019 Nov 27;8(1):139–49. doi: 10.1002/fsn3.1283 (PMC6977480; doi:10.1002/fsn3.1283)
Supplement: Supplementary file 1 [file FSN3-8-139-s001.docx]

**Supplementary materials**

**Evaluation of antimicrobial and antibiofilm properties of** **proanthocyanidins from Chinese bayberry (*Myrica rubra* Sieb. et Zucc.) leaves against *Staphylococcus epidermidis***

Table S1. Cultivable and sublethal *S*.*epidermidis* cells after EGCG or BLPs treatments at the MIC.

| **Compounds** | **non-selective media** | **selective media** | **Sublethal cells**  **(%)** |
| --- | --- | --- | --- |
|  | **TSA (log CFU/mL)** | **TSA+5% NaCl**  **(log CFU/mL)** |  |
| Control | 5.98±0.01 | 5.97±0.00 | 0.16±0.16 |
| EGCG (80 μg/mL) | 5.47±0.12 | 0.00±0.00 | 100±2.19 |
| BLPs (320 μg/mL) | 5.74±0.11 | 5.70±0.08 | 0.70±0.52 |

Table S2. Height and Roughness of *S. epidermidis* cells after EGCG or BLPs treatments at the MBIC.

| **Compounds** | **Height (nm)** | **Roughness** |
| --- | --- | --- |
| Control | 146.59±22.26^A^ | 0.83±0.02^C^ |
| EGCG (40 μg/mL) | 61.98±16.14^B^ | 15.00±7.20^B^ |
| BLPs (160 μg/mL) | 152.39±12.00^A^ | 45.53±4.15^A^ |

*Note*: Values are the mean of triplicate measurements ± standard deviation; values with different uppercase letters in the same column showed a significant difference at *p* < 0.05.

**1. Methods and Materials**

**1.1 Extraction, purification, and degradation of proanthocyanidins (PAs)**

Extraction and purification of PAs from the Chinese bayberry (*Myrica rubra* Sieb. et Zucc.) leaves were based on a previous method ([Fu et al., 2014](#_ENREF_2); [Yang et al., 2011](#_ENREF_5)). Briefly, the leaves were dried in the oven at 40 °C for 12 h and grounded into powder with the particle size around 150 μm. After the leaves powder (4 kg) was extracted with 70% acetone (40 L), the aqueous phase was obtained and washed with hexane and dichloromethane. Then, the organic solvent was evaporated by rotary evaporation and the aqueous phase was lyophilized to dryness to obtain the crude bayberry leaves proanthocyanidins extract (CBLPs). CBLPs was then purified by an HPD-500 column to remove sugar with ethanol as an elution solvent. The eluate was evaporated to dryness yielding a brown powder, which was labeled as resin purified BLPs (RPBLPs). Afterwards, RPBLPs was purified by a Sephadex LH-20 (300 mm × 30 mm i.d.) column. 90% methanol was used to elute most of the flavonoids and then 50% acetone was applied to elute most PAs and was collected. The collection was dried by rotary evaporation under vacuum to remove the organic solvent and then lyophilized to dryness to obtain SPBLPs.

**1.2 Determination of total phenolics and PAs**

The total phenolic content (TPC) was determined using the Folin–Ciocalteu assay ([Bao, Cai, Sun, Wang, & Corke, 2005](#_ENREF_1)). In brief, 400 μL of the prepared sample solution was added to 1 mL of 1 M Folin–Ciocalteu reagent. The sample was diluted to 2 mL with distilled deionized water and shaken completely. The mixture solution was maintained in the dark for 5 min at room temperature. Then, 5 mL of 5% Na_2_CO_3_ (w/v) was added to the mixture, which could stand for 60 min at room temperature. Finally, the absorbance of the mixture solution was measured at 765 nm. The results were expressed as milligrams of gallic acid equivalents per gram dried weight (DW).

The PA content was determined according to a modified vanillin assay ([Sun, Leandro, Jm, & Spranger, 1998](#_ENREF_4)). Briefly, 2.5 mL of 1% (w/v) vanillin in methanol and then 2.5 mL of 20% (v/v) H_2_SO_4_ in methanol was added to 1 mL of the prepared sample solution to perform the vanillin reaction. Then the mixture solution was kept in a 30 °C water bath for 15 min to undergo the vanillin reaction. Methanol was used as the blank. The absorbance was measured at 500 nm and compared to the prepared blank. The content of PAs was calculated based on the standard curve of EGCG.

**1.3 Liquid chromatography analysis of the mean degree of polymerization (mDP) of BLPs**

The mDP of the PAs was determined using acid catalysis in the presence of phloroglucinol based on our previous procedures ([Yang et al., 2011](#_ENREF_5)). In brief, the terminal flavan-3-ols units were cleaved by acid catalysis in the presence of phloroglucinol, while the extension units were released as phloroglucinol derivatives ([Kennedy & Jones, 2001](#_ENREF_3)). Five milligrams of PAs samples were dissolved in 1 mL of a freshly prepared methanol solution containing 0.2 N HCl, 50 g/L phloroglucinol, and 10 g/L ascorbic acid. The mixture solution was kept at 60 °C for 1 h to perform the reaction. Afterwards, 1 mL of 200 mM of sodium acetate was added to stop the reaction. The reaction mixture was then filtered through a 0.45-μm membrane and analyzed by a reverse-phase HPLC-DAD platform. The separation was performed on a Zorbax SB-C18 (Agilent, Santa Clara, CA, USA) column (250 × 4.6 mm, 5 μm) at 35 °C and was detected at 280 nm. Mobile phase A was 0.1% formic acid, and mobile phase B was methanol (flow rate: 1 mL/min). The gradient elution profile was as follows: 5% B (0-10 min), 5-20% B (10-20 min), 20-40% B (20-30 min), and 40-90% B (35-37 min).

**References**

Bao, J., Cai, Y., Sun, M., Wang, G., & Corke, H. (2005). Anthocyanins, flavonols, and free radical scavenging activity of Chinese bayberry (*Myrica rubra*) extracts and their color properties and stability. *Journal of Agricultural and Food Chemistry, 53* (6), 2327.

Fu, Y., Qiao, L., Cao, Y., Zhou, X., Liu, Y., & Ye, X. (2014). Structural elucidation and antioxidant activities of proanthocyanidins from Chinese bayberry (*Myrica rubra* Sieb. et Zucc.) leaves. *PLoS One, 9* (5), e96162.

Kennedy, J. A., & Jones, G. P. (2001). Analysis of proanthocyanidin cleavage products following acid-catalysis in the presence of excess phloroglucinol. *Journal of Agricultural and Food Chemistry, 49* (4), 1740.

Sun, B., Leandro, C., Jm, R. D. S., & Spranger, I. (1998). Separation of grape and wine proanthocyanidins according to their degree of polymerization. *Journal of Agricultural and Food Chemistry, 46* (4), 1390-1396.

Yang, H., Ye, X., Liu, D., Chen, J., Zhang, J., Shen, Y., & Yu, D. (2011). Characterization of unusual proanthocyanidins in leaves of bayberry (*Myrica rubra* Sieb. et Zucc.). *Journal of Agricultural and Food Chemistry, 59* (5), 1622-1629.
